# ω-3 PUFA Rich Camelina Oil By-Products Improve the Systemic Metabolism and Spleen Cell Functions in Fattening Pigs

**DOI:** 10.1371/journal.pone.0110186

**Published:** 2014-10-10

**Authors:** Ionelia Taranu, Mihail Gras, Gina Cecilia Pistol, Monica Motiu, Daniela E. Marin, Nicoleta Lefter, Mariana Ropota, Mihaela Habeanu

**Affiliations:** INCDBNA-IBNA, National Institute of Research and development for Biology and Animal Nutrition, Balotesti, Romania; German Institute of Human Nutrition Potsdam-Rehbrücke, Germany

## Abstract

Camelina oil-cakes results after the extraction of oil from Camelina sativa plant. In this study, camelina oil-cakes were fed to fattening pigs for 33 days and its effect on performance, plasma biochemical analytes, pro-/anti-inflammatory mediators and antioxidant detoxifying defence in spleen was investigated in comparison with sunflower meal. 24 crossbred TOPIG pigs were randomly assigned to one of two experimental dietary treatments containing either 12% sunflower meal (treatment 1-T1), or 12.0% camelina oil-cakes, rich in polyunsaturated fatty acids ω-3 (ω-3 PUFA) (treatment 2-T2). The results showed no effect of T2 diet (camelina cakes) on feed intake, average weight gain or feed efficiency. Consumption of camelina diet resulted in a significant decrease in plasma glucose concentration (18.47%) with a trend towards also a decrease of plasma cholesterol. In spleen, T2 diet modulated cellular immune response by decreasing the protein and gene expression of pro-inflammatory markers, interleukin 1-beta (IL-1β), tumor necrosis factor alpha (TNF-α), interleukin 6 (IL-6) and interleukin (IL-8) and cyclooxigenase 2 (COX-2) in comparison with T1 diet. By contrast, T2 diet increased (P<0.05) in spleen the mRNA expression of antioxidant enzymes, catalase (CAT), superoxide dismutase (SOD), and glutathione peroxidase 1 (GPx1) by 3.43, 2.47 and 1.83 fold change respectively, inducible nitric oxide synthase (iNOS) (4.60 fold), endothelial nitric oxide synthase (eNOS) (3.23 fold) and the total antioxidant level (9.02%) in plasma. Camelina diet increased also peroxisome-proliferator activated receptor gamma (PPAR-γ) mRNA and decreased that of mitogen-activated protein kinase 14 (p38α MAPK) and nuclear factor of kappa light polypeptide gene enhancer in B-cells (NF-κB). At this level of inclusion (12%) camelina oil-cakes appears to be a potentially alternative feed source for pig which preserves a high content of ω-3 PUFA indicating antioxidant properties by the stimulation of detoxifying enzymes expression and the suppression of spleen pro-inflammatory markers.

## Introduction

Nutrition and food science research is driven by increasing consumer demands for food quality and safety, and the increasing awareness of the complex relation between nutrition and health. This has led towards exploiting natural resources rich in active compounds with beneficial effects on animal and human health. Such bioactive compounds of interest are polyunsaturated fatty acids (PUFAs), especially ω-3 and ω-6 PUFAs, antioxidants, flavonoids, vitamins, and minerals. Fish oils and vegetable oils (such as linseed or rapeseed) are among the most known PUFA sources with health promoting effects on serum parameters, immune-mediators and anti-inflammatory responses, which have been widely studied in humans and animals [1]–[3]. Feeding mice with dietary fish oil, resulted in a decreased production of interleukins and tumour necrosis factor [4] while the production of IgG and IgE was enhanced by using high levels of fish oil [5]. The linseed oil in pig diet increased the proportions of long chain PUFA in the fetus and in newborns during the suckling period [6]. Also, feeding flaxseed and flaxseed meal to sows resulted in a beneficial effect on milk composition (increased protein content) and on their piglets’ post weaning growth and immune resistance (higher serum anti-ovalbumin concentration) [7]. Supplementation with rapeseed oil rich in ω-3 PUFAs has a reducing effect on cholesterol and on LDL to HDL ratio [8]. Similarly, the serum total cholesterol, HDL cholesterol, triglyceride and phospholipid concentrations were also significantly lower in senescence-accelerated male mice fed with a ω-3 PUFA-rich diet (perilla oil), compared with mice fed with a ω-6 PUFA-rich diet (sunflower oil) [9]. There is a large volume of data concerning PUFA effects and some of their sources; nevertheless, those PUFA sources are not in sufficient quantity for the food industry. Novel sources need to be investigated for nutritional and health effects. Some findings indicated flax as an excellent source of α-linolenic acid (C18∶3 ω-3), which could be used to provide dietary ω-3 PUFA with beneficial effects on animal and human health [3], [10]. Indeed, supplementation of sow diets with flax in any form (seed, meal or oil) increased ω-3 PUFA concentrations in the blood and milk of sows and in their piglets, enhancing their immune response and the post weaning growth [7], [11]. There are also other reports that flax may have an impact on immune development and growth [3], [10]. *Camelina sativa,* usually known as camelina, or occasionally “wild flax” or “false flax”, has a similar fat composition to flaxseed [12], and appears to be a potential alternative source to flax, and has been investigated for its exceptionally high levels of ω-3/ω-6 fatty acids (35–40%), protein (35–40%) and γ-tocopherol [13]–[15]. This crop recently became attractive as a biofuel source; its oil is cheaper than the oil from other crops [13], [16]. Camelina by-products (i.e. meal/oil-cakes) obtained after oil extraction from the seed are an important co-product of considerable nutritional value [16], being rich in protein, essential amino acids, fat and essential ω-3 and ω-6 fatty acids. Several nutritional researches reported the possibility of using camelina oil and its by-products in the diet of animals [17]–[19]. Consumption of eggs from hens fed on diets containing camelina meal could provide more than 300 mg/day of ω-3 fatty acids to the human diet [14]. Diets including either camelina seed (630 g/d, CS diet), or camelina meal (2 kg/d, CM diet) generated a higher proportion of monounsaturated fatty acids in the milk of Holstein dairy cows, although they led to a decrease in milk fat content [20]. In pigs, especially piglets, the effect of ω-3 and ω-6 PUFA has not been extensively investigated irrespective of the sources [1]. The dietary addition of ω-3 PUFA could have a beneficial effect by inhibiting the production of inflammatory substances [1]. Furthermore, in pigs, little is known about the effect of camelina (oil or oil-cakes) as an alternative source of ω-3 PUFA, and studies are necessary to adjust their dietary rate inclusion specifically for this species [16], [19]. It was considered that camelina could rise nutritional problems because of its content in erucic acid and glucosinolates, but recent studies have demonstrated the chemo-protective and anti-cancer effect of glicosinolates [19].

In the present study, the potential of 12% dietary camelina oil-cakes was investigated in fattening and finishing pigs which are also used as an animal model for the assessment of immune, metabolic and general health status. The effect on inflammatory mediators, signaling pathway molecules and antioxidant-detoxifying enzymes in the spleen and plasma was compared with that produced by sunflower meal, used as control.

## Material and Methods

### 1. Experimental design. Animals, diet and sampling

A total of 24 crossbred TOPIG hybrid pigs with an average body weight of 68.45±3.5 kg were divided into two experimental groups with 12 pigs/group, housed in pens and fed with wheat-barley isoenergetic and isoproteic diets, containing 12% sunflower meal (control treatment T1), and 12% camelina oil-cakes (experimental treatment T2) for 33 days (Table 1). The camelina used to obtain the cakes was produced in the field of Romanian Agricultural Institute Fundulea from a Romanian variety of *Camelina sativa* “Camelia” and kindly provided by Dr. Toncea after cold pressure oil extraction. The respective treatments were formulated to meet all nutritional requirements of growing fattening pigs (NRC, 1988). Pigs were given *ad libitum* access to water and feed, and were individually weighed at the beginning and of the end of trial. Feed consumption was recorded daily per pen. The average daily gain (ADG), average daily feed intake (ADFI), and gain-feed ratio (G:F) were calculated. At the end of the trial (33d) blood samples from 12 pigs/group were aseptically collected into 9-mLVacutainer tubes containing 14.3 U/mL of lithium heparin (Vacutest, Arzergrande, Italy) and centrifuged at 775×*g* for 25 min at 4°C. The resulting plasma was used to determine immunoglobulin and cytokines concentrations, the plasma biochemical parameters and the antioxidant capacity. Pigs did not receive feed before blood sample collection. Animals were sacrificed at 33d and organ samples were collected on ice; spleen aliquots (30–50 g) were stored at –80°C until analyzed. Animals were observed twice daily, and cared for in accordance with the Romanian Law 206/2004 for handling and protection of animals used for experimental purposes and according to EU Council Directive 98/58/EC concerning the protection of farmed animals. All efforts were made to minimize suffering. The study protocol was approved by the Ethical Committee of the National Research-Development Institute for Animal Nutrition and Biology, Balotesti, Romania.

**Table 1:** Composition and calculated nutrient content of experimental diets (%).

	Finishing phase^1^	
Items	T1	T2
Corn	52.84	42.08
Barley	10.00	16.00
Rice meal	12.00	17.00
Soybean meal (44%)	8.00	9.00
Sunflower meal (31.94%)	12.00	-
Camelina cakes	-	12.00
Sunflower oil	1.00	0.20
L-lysine-HCl (80%)	0.32	0.20
Methionine (99%)	0.02	-
Limestone	1.57	1.62
Monocalcium phosphate	0.75	0.42
NaCl	0.40	0.40
Choline premix	0.10	0.10
Mineral vitamin-premix^2^	1.00	1.00
Calculated Nutrient content		
CP (%)	14.63	14.94
ME (Kcal/kg)	3058	3057
Lysine (%)	0.87	0.88
Digestible Lysine (%)	0.74	0.76
Met + Cys (%)	0.59	0.60
Digestible Met + Cys (%)	0.48	0.47
Calcium (%)	0.80	0.80
Phosphorus (%)	0.65	0.65
Crude fibre (%)	5.16	4.39

1 BW range 68.45 to 98.0 kg.

2 mineral-vitamin premix (1%) supplied per kg diet as follows: vit. A 6000 IU, vit. D3 800 IU, vit. E 20 IU, vit. K1 1.0 mg, vit. B_1_ 1.0 mg, vit. B_2_ 3.0 mg, d-pantothenic acid 6.3 mg, niacin 10 mg, biotin 30 µg. vit. B_12_ 20 µg, folic acid 0.3 mg, vit. B_6_ 1.5 mg, Fe 80 mg, Zn 25 mg, Mn 30 mg, I 0.22 mg. Se 0.22 mg, Co 0.3 mg, antioxidants 60 mg. and maize starch as carrier.

### 2. Dietary fatty acid analysis

Feed samples were taken at the beginning of the experiment, and were analyzed for fatty acid composition. Lipids were extracted by the methanol-hexane procedure (ASRO-SR EN ISO 15304/AC, 2005). The samples were analyzed by using a Perkin Elmer gas chromatograph (Clarus 500, USA) equipped with injector (250°C temperature), flame-ionization detector (260°C temperature), and BPX70 capillary chromatographic column for fatty acid methyl esters (60 m×0.25 mm i.d.×0.20 µm, Agilent, column flux was 50 mL/min, and the split ratio was 1∶100). The temperature program was as follows: increase from 180°C to 220°C at 5°C/min and maintain for 7 min, then increase to 220°C at 5°C/min and maintain for 10 min. The total analysis time was 29 min. Peaks were identified by comparing their retention times with individual reference standard fatty acids solution (methylated 37 Component FAMWE Mix, SUPELCO, USA and Soybean oil, SUPELCO, USA) (Table 2 and Table 3).

**Table 2:** Fatty acid composition (g/100 g of fatty acids) of sunflower meal and camelina oil cakes.

Items	Sunflower meal	Camelina oil-cake
Miristic acid (C14∶0)	0.13	0.10
Palmitic acid (C16∶0)	9.52	7.09
Stearic acid (C18∶0)	3.34	2.04
Arahidic acid (C20∶0)	0.05	1.08
Palmitoleic (C16∶1n-7)	0.19	0.19
Oleic cis acid (C18∶1n-9)	27.03	15.57
Eicosanoic acid (C20∶1n-9)	0.00	9.91
Erucic acid (C22∶1n-9)	0.00	1.95
Linoleic acid (C18∶2n-6)	58.34	24.96
Conjugated Linoleic acid (CLA)	0.35	0.00
Eicosadienoic acid (C20∶2n-6)	0.00	1.73
Arachidonic acid (C20∶4n-6)	0.00	1.00
α -Linolenic (C18∶3n-3)	0.16	31.50
Octadecatetraenoic (C18∶4n-3)	0.00	1.02
Eicosapentaenoic (C20∶5n-3)	0.00	0.23
Docosapentaenoic (C22∶5n-3)	0.00	0.10
Other fatty acids	0.89	0.69
Total		
Σ Saturated acids	13.04	10.31
Σ Unsaturated acids	86.07	88.16
Σ n-6	58.69	28.02
Σ n-3	0.16	33.36
Ratio n-6/n-3	366.81	0.84
Linoleic/α -Linolenic	364.63	0.79

**Table 3:** Fatty acid composition of experimental diets (g/100 g of total fatty acids).

Items	Finishing phase^1^	
	T1	T2
Miristic acid (C14∶0)	0.17	0.22
Palmitic acid (C16∶0)	13.95	14.44
Stearic acid (C18∶0)	2.24	1.90
Arahidic acid (C20∶0)	0.40	0.60
Palmitoleic (C16∶1n-7)	0.16	0.24
Oleic acid (C18∶1n-9)	31.18	29.74
Eicosanoic acid (C20∶1n-9)	0.33	2.26
Erucic acid (C22∶1n-9)	0.00	0.48
Linoleic acid (C18∶2n-6)	49.62	43.28
Eicosadienoic acid (C20∶2n-6)	0.00	0.26
Arachidonic acid (C20∶4n-6)	0.00	0.11
α -Linolenic (C18∶3n-3)	1.50	5.65
Octadecatetraenoic (C18∶4n-3)	0.00	0.23
Eicosapentaenoic (C20∶5n-3)	0.25	0.33
Docosapentaenoic (C22∶5n-3)	0.20	0.19
Other fatty acids	0.00	0.00
**Total**		
Σ Saturated acids	16.76	17.16
Σ Unsaturated acids	83.24	82.77
Σ n-6	49.62	43.65
Σ n-3	1.95	6.40
Ratio n-6/n-3	25.45	6.82
Linoleic/α -Linolenic	33.08	7.66

1 BW range 68.45 to 98.0 kg.

T1: diet containing 12% sunflower meal.

T2: experimental diet including 12% camelina oil-cakes.

### 3. Measurement of plasma biochemical parameters

Concentration of glucose, total cholesterol, high-density lipoprotein cholesterol (HDL cholesterol), triglycerides, total protein, urea, Ca, Mg, Fe and the activity of alkaline phosphatase (ALKP), glutamate pyruvate transaminase (TGP), and glutamate oxaloacetate transaminase (TGO) were determined on an automatic BS-130 Chemistry analyzer (Bio- Medical Electronics Co., LTD, China), on plasma of blood collected at the end of the experiment, and then centrifuged for 25 minutes at 3500×*g*.

### 4. Measurement of total plasma immunoglobulin subsets (IgG, IgA, IgM)

The total concentration of immunoglobulin (Ig) subsets (G, A and M) was measured by ELISA (Bethyl, Medist, Montgomery, TX, USA) in blood plasma, after plasma dilution, as follows: 1∶4000 (IgA), 1∶120,000 (IgG), and 1∶10,000 (IgM), as reported previously [21], according to the manufacturer’s instructions. Absorbance was read at 450 nm using a microplate reader (Tecan Sunrise, Austria).

### 5. Measurement of spleen antioxidant capacity

Antioxidant level in spleen tissue of pigs fed with control sunflower diet or with camelina oil-cakes was measured with the total antioxidant capacity (TAC) kit (QuantiChrom – BioAssay Systems, USA). Briefly, frozen spleen tissue samples (100 mg) were disrupted and homogenized by using Ultra-Turrax homogenizer (IKA-Werke GmbH & Co. KG, Germany) and phosphate buffer containing IGEPAL 1%, sodium deoxycholate 0.5%, SDS 0.1% and complete (EDTA-free) protease inhibitor cocktail tablets. The homogenates were kept 30 min on ice, and then centrifuged at 10,000×g at 4°C for 10 min. 20 µL of spleen tissue lysate or Trolox standard solution plus 100 µL Working Reagent were added to 96-well microplate, mixed by tapping and incubated at room temperature for 10 minutes according to the manufacturer’s instructions. The end point absorbance was read at 570 nm using a microplate reader (TECAN, Sunrise, Austria).

### 6. Measurement of spleen nitric oxide production

Nitric oxide (NO) level in spleen of pigs fed with control sunflower diet or with camelina oil-cakes was measured to determine the synthesis of NO by Griess assay as previously described [22]. After protein precipitation, 100 µL of the supernatant were mixed with an equal volume of Griess reagent (1% sulfanilamide, 0.1% naphthylethylene diamine dihydrochloride and 2.5% phosphoric acid) and incubated at 37°C for 10 minutes. Nitrite absorbance was measured at 550 nm using a microplate reader (TECAN, Sunrise, Austria) and a NaNO_2_ standard curve ranging from 0 to 100 µM. Concentration was calculated based on the NaNO_2_ range, expressed as µmole/L NO_2_^−^.

### 7. Analysis of gene expression (qPCR)

#### 7.1 Extraction of total RNA

Frozen spleen tissue samples (100 mg) were disrupted and homogenized in RTL buffer (QIAGEN GmbH, Germany) using Ultra-Turrax homogenizer (IKA-Werke GmbH & Co. KG, Germany). Total RNA was extracted using Qiagen RNeasy midi kit (QIAGEN GmbH, Germany), according to the manufacturer’s recommendations. After extraction, RNA was treated with a ribonuclease inhibitor (RNasin Plus RNase Inhibitor; Promega Corp., USA) and the quantity and quality of extracted total RNA were measured on a Nanodrop ND-1000 spectrophotometer (Thermo Fischer Scientific, USA). The integrity of RNA was verified by agarose gel electrophoresis.

#### 7.2 cDNA synthesis

After extraction of total RNA from each spleen sample cDNA was generated using M-MuLV Reverse Trascriptase kit (Fermentas, Thermo Fischer Scientific, USA) according to the manufacturer’s protocol. Briefly, 1 µg of total RNA was used as starting material, to which 0.5 µg of oligo (dT) was added. RNAs and oligo (dT) were mixed gently, then centrifuged and incubated at 65°C for 5 min, chilled on ice, centrifuged and placed on ice again. 4 µL of 5X reaction buffer, 2 µLof dNTP Mix (1 mM each dNTP) and 2 µL of MMuLV Reverse Transcriptase (40 U) were added further to the mix. The samples were incubated at 42°C for 60 min, and the reaction was inactivated at 70°C for 10 min.

#### 7.3 Quantitative Real-Time PCR

Fluorescent real-time PCR was used to evaluate the pro- and anti-inflammatory markers (TNF-α, IL-1β, IL-6, IL-8, IFN-γ, IL-4, COX2, iNOS, eNOS), antioxidant enzymes (SOD, CAT, GPx1), and signaling molecules (PPAR-γ, MAPK p-38α, NF-κB) gene expressions. Reactions were set up in a total volume of 20 µl using 5 µl of cDNA (diluted 1∶10), 12.5 µl Maxima SYBR Green/Fluorescein qPCR Master Mix 2X (Fermentas, Thermo Fischer Scientific, USA), 0.3 µM each of gene-specific primer (Table 4) and performed in the Rotor-Gene-Q (QIAGEN GmbH, Germany) machine. The cycling conditions were: UDG pre-treatment at 50°C for 2 min, initial denaturation step at 95°C for 15 s, followed by 40 cycles of 95°C for 15 s, 60°C for 15 s and 72°C for 15 s with a single fluorescence measurement; a final elongation step was carried out at 72°C for 10 min. The specificity of the PCR products was confirmed by analysis of the dissociation curve. The melting curve program consisted of temperatures between 60 and 95°C with a heating rate of 0.1°C/s and a continuous fluorescence measurement. All samples were measured in triplicate. The relative product levels were quantified using the 2^(−ΔΔ C^_q_^)^ method [23]. The average level of expression of two reference genes, Cyclophilin A and βactin were used for data normalisation. These reference genes were experimentally validated for IPEC-1 type cell and the lack of treatment effect and of expression variation was the selection criteria for the reference genes. The results were expressed as relative fold increase or decrease from untreated control cells.

**Table 4:** Nucleotide sequences of primers for Real-Time PCR.

Gene	Accesion no.	Primer source	Primer sequence (5′→3′)	Orientation	Tm (°C)	Amplicon lenght (bp)	References
TNF-α	NM_214022	Pig	ACTGCACTTCGAGGTTATCGG	forward	60	118	[69]
			GGCGACGGGCTTATCTGA	reverse	60		
IL-8	NM_213867.1	Pig	GCTCTCTGTGAGGCTGCAGTTC	forward	58	79	[69]
			AAGGTGTGGAATGCGTATTTATGC	reverse	54		
IL-6	NM_214399	Pig	GGCAAAAGGGAAAGAATCCAG	forward	57	87	[69]
			CGTTCTGTGACTGCAGCTTATCC	reverse	61		
IL-1β	NM_214055	Pig	ATGCTGAAGGCTCTCCACCTC	forward	62	89	[70]
			TTGTTGCTATCATCTCCTTGCAC	reverse	59		
IFN-γ	NM_213948.1	Pig	TGGTAGCTCTGGGAAACTGAATG	forward	54	79	[70]
			GGCTTTGCGCTGGATCTG	reverse	55		
GAPDH	NM_001206359.1	Pig	ACTCACTCTTCTACCTTTGATGCT	forward	57	100	[71]
			TGTTGCTGTAGCCAAATTCA	reverse	55		
IL4	NM_214123.1	Pig	CAACCCTGGTCTGCTTACTG CTTCTCCGTCGTGTTCTCTG	Forward reverse	5252	173	[72]
CAT	NM_214301.2	Pig	CTTCTCCGTCGTGTTCTCTG	forward	55	241	[73]
			GTCCAGAAGAGCCTGAATGC	reverse	55		
COX2	NM_214321.1	Pig	CCCAATTTGTTGAATCATTT	forward	55	119	[74]
			TCTCATCTCTCTGCTCTGGT	reverse	55		
GPx	NM_214201.1	Pig	GGAGATCCTGAATTGCCTCAAG	forward	56	62	[75]
			GCATGAAGTTGGGCTCGAA	reverse	57		
iNOS	NM_001143690.1	Pig	GGAGCCATCATGAACCCCAA	forward	60	73	[76]
			GTAGAAGCTCGTCTGGTGGG	reverse	62		
SOD	NM_001190422.1	Pig	GAGACCTGGGCAATGTGACT	forward	62	139	[76]
			CTGCCCAAGTCATCTGGTTT	reverse	58		
PPARγ	NM_214379.1	Pig	ACTGTCGGTTTCAGAAGTGC	forward	53	138	[76]
			CAGCAGACTCTGGGTTCAGT	reverse	53		
p38α-MAPK	XM_003356616.1	Pig	TGCAAGGTCTCTGGAGGAAT	forward	52	109	[77]
			CTGAACGTGGTCATCCGTAA	reverse	52		
NF-kB	NM_001114281.1	Pig	CGAGAGGAGCACGGATACCA	forward	55	62	[78]
			GCCCCGTGTAGCCATTGA	reverse	54		
Cyclophilin A	NM_214353.1	Pig	CCCACCGTCTTCTTCGACAT	forward	54	92	[79]
			TCTGCTGTCTTTGGAACTTTGTCT	reverse	55		
Beta-2 microglobulin	NM_213978.1	Pig	TTCTACCTTCTGGTCCACACTGA	forward	55	162	[80]
			TCATCCAACCCAGATGCA	reverse	50		

### 8. Cytokine protein detection (ELISA)

Cytokine (IL-8, IL1-β, IL-6, TNF-α, IL-4, and IFN-γ) concentration was measured in both blood plasma and spleen (d33). Samples of spleen tissue were weighed and homogenized in phosphate buffer containing IGEPAL 1%, sodium deoxycholate 0.5%, SDS 0.1% and complete (EDTA-free) protease inhibitor cocktail tablets. The homogenates were kept 30 min on ice, and then centrifuged at 10,000×g at 4°C for 10 min. Plasma samples were used undiluted for ELISA detection. Cytokine concentrations in the tissue supernatants and plasma were determined by ELISA, using commercially available kits (R&D Systems, Minneapolis, MN 55413, USA), according to the manufacturer’s instructions. Purified fractions of anti-swine cytokines IL-8 (MAB5351), TNF-α (MAB6902), IL-1β (MAB6811), IL-6 (MAB686), IL-4 (ASC0944) and IFN-γ (ASC4934) (R&D Systems, Minneapolis, USA and Biosource International, Inc., Camarillo, USA) were used as capture antibody, in conjunction with biotinylated anti-swine cytokines IL-8 (BAF535), TNF-α (BAF690), IL-1β (BAF681), IL-6 (BAF686), IL-4 (ASC0849), IFN-γ (ASC4839). Streptavidin-HRP (Biosource, Camarillo, USA) and TMB (tetramethylbenzidine) was used for detection. Optical densities were measured on an ELISA microplate reader (Tecan, SunRise, Austria) at 450 nm. Dilutions of recombinant swine IL-8, TNF-α, IL-1β, IL-4 and IFN-γ were used as standards, and data wasanalyzed against the linear portion of the generated standard curve. Results were expressed as picograms (pg) of cytokine/mL of plasma or pg of cytokine/1 mg spleen protein. Total spleen protein was quantified using bovine serum albumin as standard (Pierce BCA Protein Assay Kit, Thermo Fischer Scientific, USA).

### 9. Immunobloting analyses

The levels of phosphorylated MAPK-p38α and NF-κB p65 protein expression were analysed by Western immunoblot using rabbit anti-porcine phospho-MAPK-p38 (Thr180/Tyr182) and rabbit anti-porcine phospho-NF-κB p65 antibodies (Cell Signaling Technology, Danvers, MA, USA) diluted 1∶200. Rabbit anti-β-actin antibody (Cell Signaling Technology), diluted 1∶500 was used as control. Spleen lysates were obtained from 2 g of frozen spleen homogenized in RIPA buffer for phosho-MAPK-p38α as previously described [24] and in RIPA-modified phosphate buffer for phospho-NF-κB [25] and were quantified for total protein content, using a commercial kit (Pierce BCA Protein Assay Kit, Thermo Fischer Scientific, USA). 30 µg of total proteins were then separated on 10% SDS-PAGE and transferred onto nitrocellulose membranes. Membranes were blocked overnight with Tris Buffer Saline (pH 7.5), 5% BSA and probed with primary antibody for 2 hours at room temperature. After washing five times with TBS containing 0.1% Tween 20 (TBST), blots were incubated with 1∶2000 horseradish-conjugated IgG secondary antibody (Cell Signaling Technology) for 1 hour and washed with TBST three more times. Protein expression was detected using ECL chemiluminescent substrate (Bio-Rad Laboratories, USA) according to the manufacturer’s instructions. Signal intensities were estimated using a chemiluminiscence MicroChemi 4.2 chemiluminescence imager (DNR Bio-Imaging Systems Ltd., Israel) and GelQuant 1D software (DNR Bio-Imaging Systems Ltd., Israel). The results were expressed as a ratio between the phosphorylation level of p38-MAP kinase and NF-κB and the expression level of β-actin.

### 10. Statistical analyses

All data are expressed as mean ± standard error of the mean (SEM). One way ANOVA analysis was performed to investigate the statistical differences between groups for all parameters analysed. Further differences between means were determined by the least square difference Fisher procedure. The statistical analysis of the data was carried out with Statview software 5.0 (SAS Institute Inc, Cary, NC) and values of P<0.05 were considered significant. The Pearson correlation coefficient was used to establish the relationships between gene expression (g_1_, g_2_) of nuclear receptors PPARγ and NF-κB, MAPK-p38α signaling, inflammation-related molecules and antioxidant defense enzymes in the spleen of pigs treated with dietary camelina oil-cakes. Statistical analysis was performed with R software (http://www.r-project.org/).

## Results

### 1. Dietary fatty acid composition

The fatty acid composition presented in Table 2 shows a higher content of ω-3 PUFA in camelina oil-cakes in comparison with sunflower meal, 33.36 vs. 0.16 (Table 2). The dietary inclusion of camelina oil-cakes increased the ω-3 PUFA content of the experimental diet (6.40 vs 1.95) and resulted in a decrease of the ω-6/ω-3 PUFAs ratio from 25.45 to 6.82 (Table 3).

### 2. Performance

Pigs fed with T1 or T2 diets for 33 days appeared clinically normal during the whole experimental period. At the end of the feeding trial, neither the average daily gain, (0.866 kg/pig/day T1 group *vs* 0.836 kg/pig/day T2 group, P = 0.592), nor the daily feed intake (3.31 kg/pig/day T1 group *vs* 3.03 kg/pig/day T2 group, P = 0.456), nor the feed:gain ratio (3.82 *vs* 3.62, P = 0.696) were influenced by the dietary treatments (data not shown).

### 3. Effect of camelina oil-cakes on plasma biochemical profile and immunoglobulin concentration

Pigs on the camelina oil-cakes diet had a significant decrease (18.47%) of plasma glucose concentration (68.68 mg/dL) compared with those on the T1 diet (84.24 mg/dL, P = 0.018). Other plasma biochemistry constituents as well as the concentration of non-specific immunoglobulin subsets (IgM, IgA, IgG) were not affected by camelina dietary treatment; the observed differences were insignificant in comparison with the T1 group. However, a trend towards a decrease in cholesterol concentration, though not statistically significant for the duration of this 33d experiment, was identified in the plasma of pigs receiving the diet with camelina oil-cakes (Table 5 and Table 6).

**Table 5:** Effects of T1 diet (sunflower meal) or T2 diet (camelina oil cakes) on selected blood biochemical parameters*.

	Treatments			
Items	T1	T2	*SEM*	*P-value*
Glucose (mg/dL)	84.24^a^	68.68^b^	2.70	0.018
Total Cholesterol (mg/dL)	82.28	78.85	1.87	0.372
Triglycerides (mg/dL)	26.98	29.81	0.96	0.144
Calcium (mg/dL)	11.93	11.78	0.18	0.692
Magnesium (mg/dL)	2.40	2.60	0.13	0.463
Total protein (mg/dL)	7.74	7.34	0.12	0.087
Albumina (g/L)	3.96	3.89	0.09	0.666
Bilirubin (mg/dL)	0.06	0.07	0.04	0.159
Urea (mg/dL)	16.25	15.36	0.90	0.635
Creatinine (mg/dL)	1.67	1.68	0.042	0.923
ALKP (IU/L) U/L	73.67	73.52	3.318	0.985
TGO (IU/L) U/L	44.35	53.10	3.419	0.209
TGP (IU/L) U/L	47.41	46.75	2.131	0.881
Gamma GT (U/L)	29.13	29.86	2.430	0.457

*Pigs received two different dietary fat treatments: T1 diet (12% sunflower meal) and T2 (12% camelina oil cakes) diet for 33d. At the end of the experiment plasma from 12 pigs/group was used to measure the blood biochemical parameters. Data are means ± standard error of the mean (SEM).

a,b = Mean values within a row with unlike superscript letters were significantly different (P<0·05).

**Table 6:** Effects of T1 diet (sunflower meal) or T2 diet (camelina oil cakes) on on different plasma immunoglobulin subsets*.

	Treatments			
Items**	T1	T2	*SEM*	*P-value*
IgA (mg/mL)	2.03	2.12	0.159	0.581
IgM (mg/mL)	3.53	3.79	0.193	0.505
IgG (mg/mL)	9.02	8.94	0.472	0.926

*Pigs received two different dietary fat treatments: T1 diet (12% sunflower meal) and T2 (12% camelina oil cakes) diet for 33d. At the end of the experiment plasma from 12 pigs/group was used to measure the plasma Ig concentration.

**Results are expressed as Ig A, M, or G content in the plasma of pigs, mean ± SEM.

### 4. Effect of camelina oil-cakes on spleen antioxidant capacity and nitric oxide synthesis

**Figure 1:**
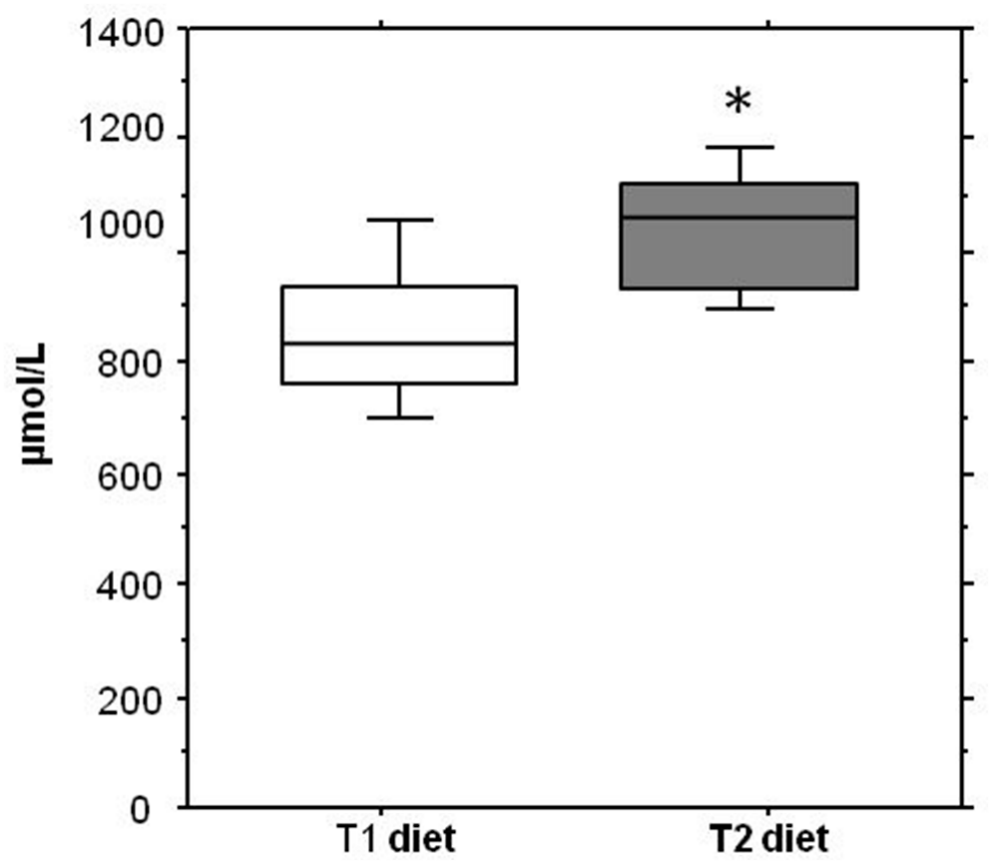
Effect of camelina oil-cakes on the antioxidant capacity in spleen. The antioxidant level in spleen samples derived from pigs fed with camelina oil-cakes or control was measured as antioxidant capacity (TAC) kit (QuantiChrom – BioAssay Systems, USA). Results are expressed as Trolox equivalent antioxidant capacity. Data are means ± SEM. ANOVA one-way test followed by Fisher test was performed to analyze the effect of the different treatments on TEAC level (*P<0.05, T1 diet-control group (white column) versus T2 diet -Camelina group (gray column).

**Figure 2:**
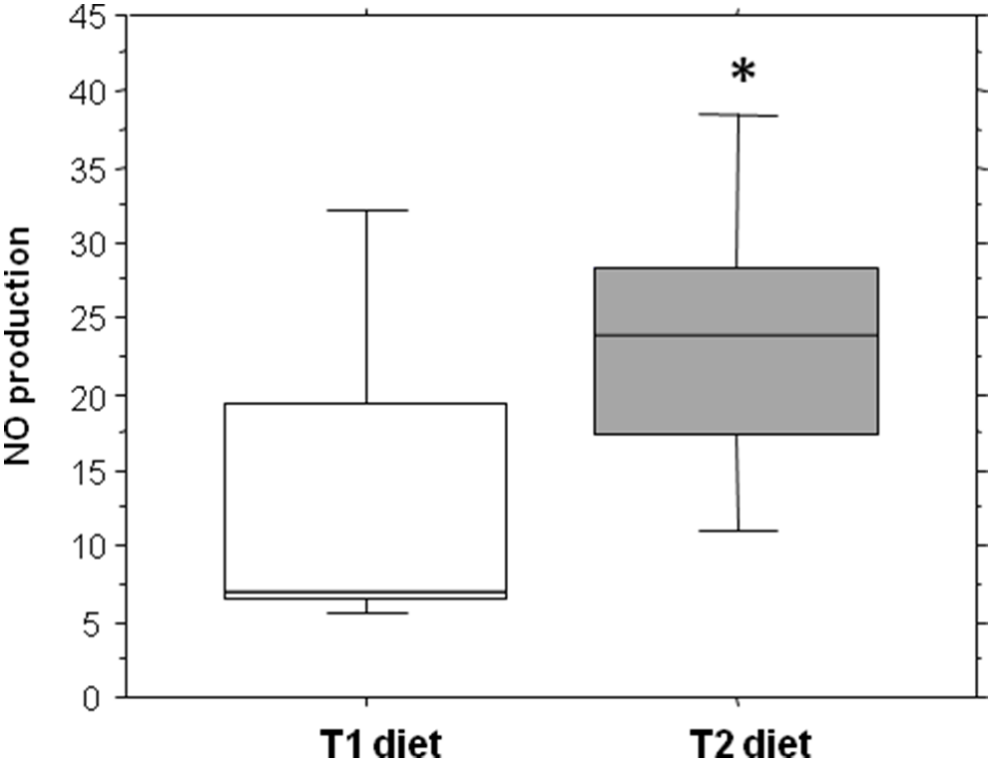
Effect of camelina oil-cakes on NO production in spleen. Synthesis of NO was determined by measuring the nitric oxide level in spleen of pigs fed or not with camelina oil-cakes using the Griess assay. Nitrite absorbance was measured at 550 using a microplate reader (Tecan Infinite 200Pro, Austria) and a NaNO_2_ standard curve ranging from 0 to 100 µM. Concentrations were calculated based on the NaNO_2_ range, expressed as µmole/L NO_2_^−^. Values are the means ± SEM, from two replicates. Statistical analysis was performed using one-way ANOVA followed by Fisher test (*P<0.05, T1 diet-control group (white column) versus T2 diet -Camelina group (grey column).

The effect of the camelina oil-cakes diet on total antioxidant status and NO production in the spleens of pigs was assessed. The results showed a statistically significant increase of the total TAC (1060.35 *vs* 911.55, P = 0.002) and NO (47.31 *vs* 28.7, P = 0.053) in the spleens of pigs treated for 33d with the camelina oil-cakes diet (Figure 1 and Figure 2).

### 5. Effect of the dietary camelina oil-cakes on protein concentration and mRNA gene expression of inflammatory markers in spleen and plasma

**Figure 3:**
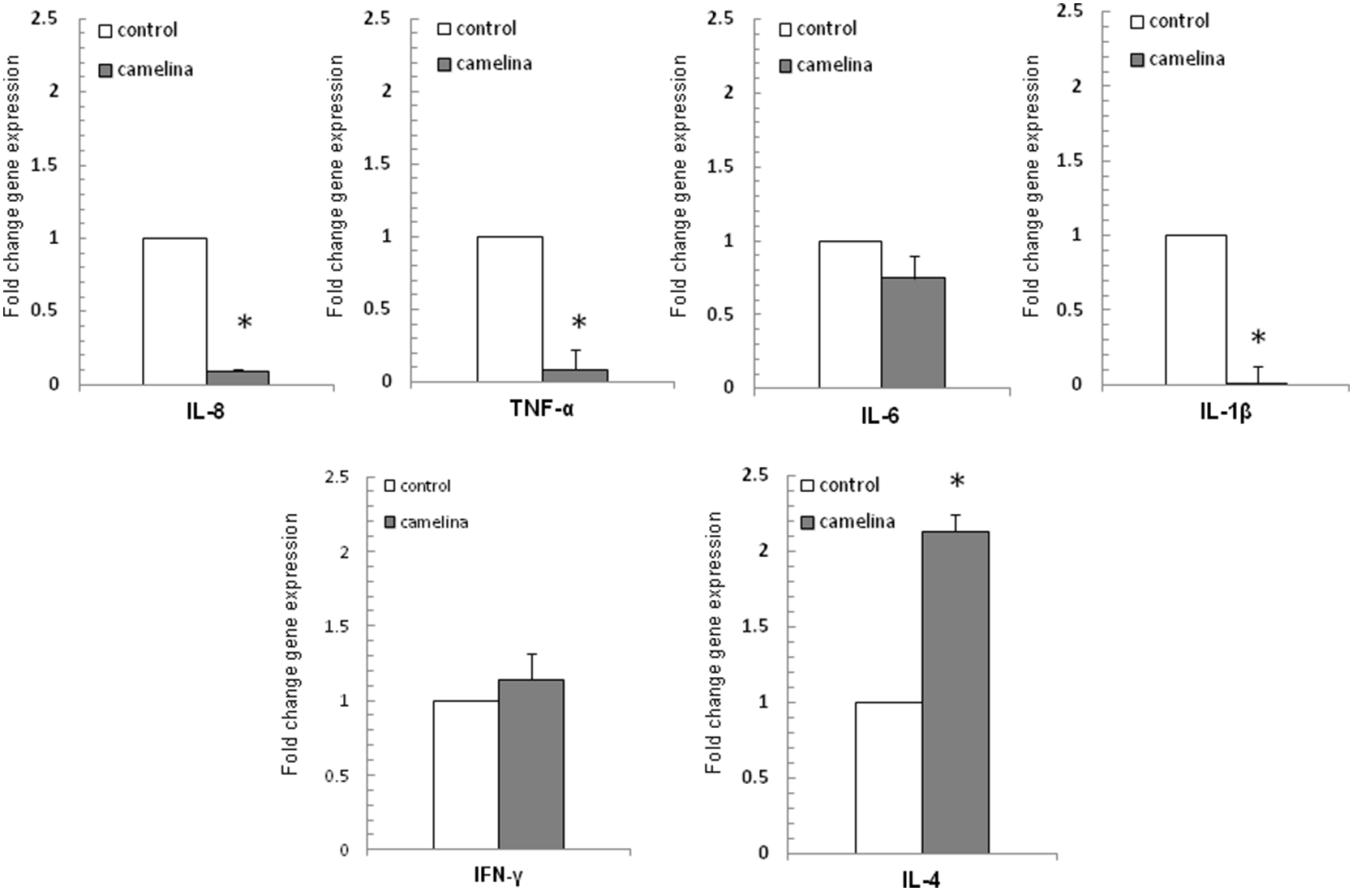
Effect of camelina oil-cakes on spleen cytokines expression. Pigs received two different dietary fat treatments: T1 (sunflower oil) and T2 (camelina oil-cakes) diet. Spleen samples were taken on day 33 of treatments and were analyzed for cytokine mRNA expression by quantitative RT-PCR. Results are expressed as fold change after normalization of the expression of target cytokine gene to the mean of 2 internally expressed reference genes. Values are the means ± SEM, from two replicates. Statistical analysis was performed using one-way ANOVA followed by Fisher test (*P<0.05, T1 diet-control group (white column) versus T2 diet -Camelina group (grey column).

**Figure 4:**
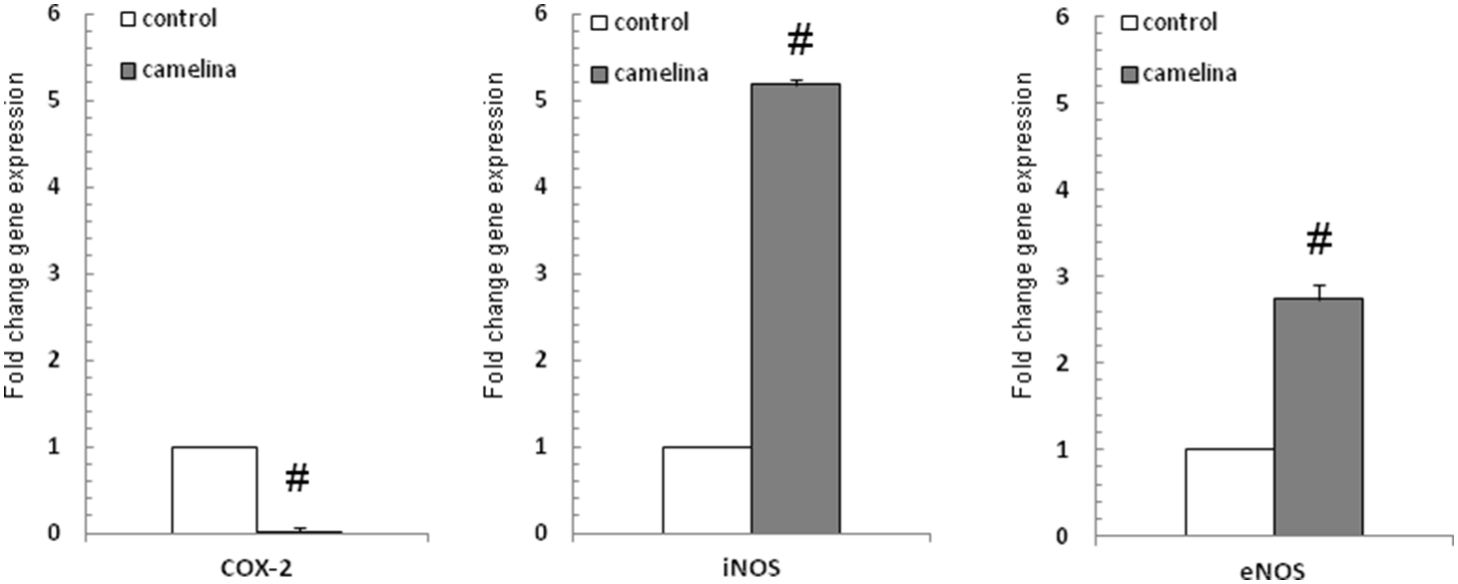
Effect of camelina oil-cakes on inflammatory markers expression in spleen. Spleen samples were taken at the end of the trial on day 33 and were analyzed for COX-2, iNOS and eNOS mRNA expression by quantitative RT-PCR. Results are expressed as fold change after normalization of the expression of target gene to the mean of 2 internally reference genes expression. Values are the means ± SEM, from two replicates. Statistical analysis was performed using one-way ANOVA followed by Fisher test (*P<0.05, T1 diet-control group (white column) versus T2 diet -Camelina group (grey column).

The ability of the camelina oil-cakes diet to modulate cytokine gene expression and cytokine production was investigated in the spleen and plasma after 33d of treatment. Regulatory (IL-4 and IFN-γ) and pro-inflammatory (TNF-α, IL-8, IL-6, IL-1β) cytokines were measured by qPCR and ELISA. As shown in Figure 3, the camelina oil-cakes diet induced a decrease (P<0.05) in TNF-α, IL-8, IL-1β and IL-6 mRNA compared to the T1 diet, and had no effect on IFN-γ. The qPCR results for other inflammation mediators showed that the camelina oil-cakes diet also resulted in a significant (P<0.00001) decrease in COX2 mRNA and an increase in iNOS and eNOS mRNA (5.18 and 2.74 times respectively) (Figure 4). Contrary to this, the expression of mRNA encoding for IL-4 increased significantly (2.32 times) in the spleen samples from pigs on the camelina oil-cakes diet (Figure 3). As expected, a similar effect on the profile of cytokines (TNF-α, IL-8, IL-1β and IL-6) at the protein level was identified in spleen with the exception of IL-4 concentration (Table 7). At a systemic level, only IFN-γ and IL-4 were detectable in plasma; the camelina oil-cakes diet increased IL-4 protein concentration and induced a slight decrease in IFN-γ cytokines (Table 7).

**Table 7:** Cytokine concentrations* in plasma and spleen of pigs fed T1 diet (sunflower meal) or T2 diet (camelina oil cakes).

	Treatment			
Cytokines	T1	T2	SEM	*p-value*
Plasma (pg/mL)				
IL-4	12.02	15.93	0.351	0.340
IFN-γ	1976.25	1851.58	176.95	0.856
Spleen (pg/mg potein)				
IL-1β	11.90	8.88	1.050	0.155
IL-8	29.21	22.72	0.399	0.168
TNF-α	23.43^a^	11.07^b^	3.680	0.094
IL-6	18.14^a^	12.68^b^	1.092	0.009
IFN-γ	3.79	3.46	0.299	0.857
IL-4	4.08	3.65	0.166	0.127

*Concentration of cytokines was measured by ELISA in samples of spleen and plasma collected at the end of the experiment, using R&D Systems kits (according to the manufacturer’s instructions). Results were expressed as picograms (pg) of cytokine/mg of spleen protein or/ml of plasma. Data are means ± SEM (n = 12).

a,b,c = Means with different superscripts within a row are significantly different (P<0.0).

### 6. Effects of the dietary camelina oil-cakes on the gene expression of PPARγ, MAPK-p38α and NF-κB signaling molecules in spleen

**Figure 5:**
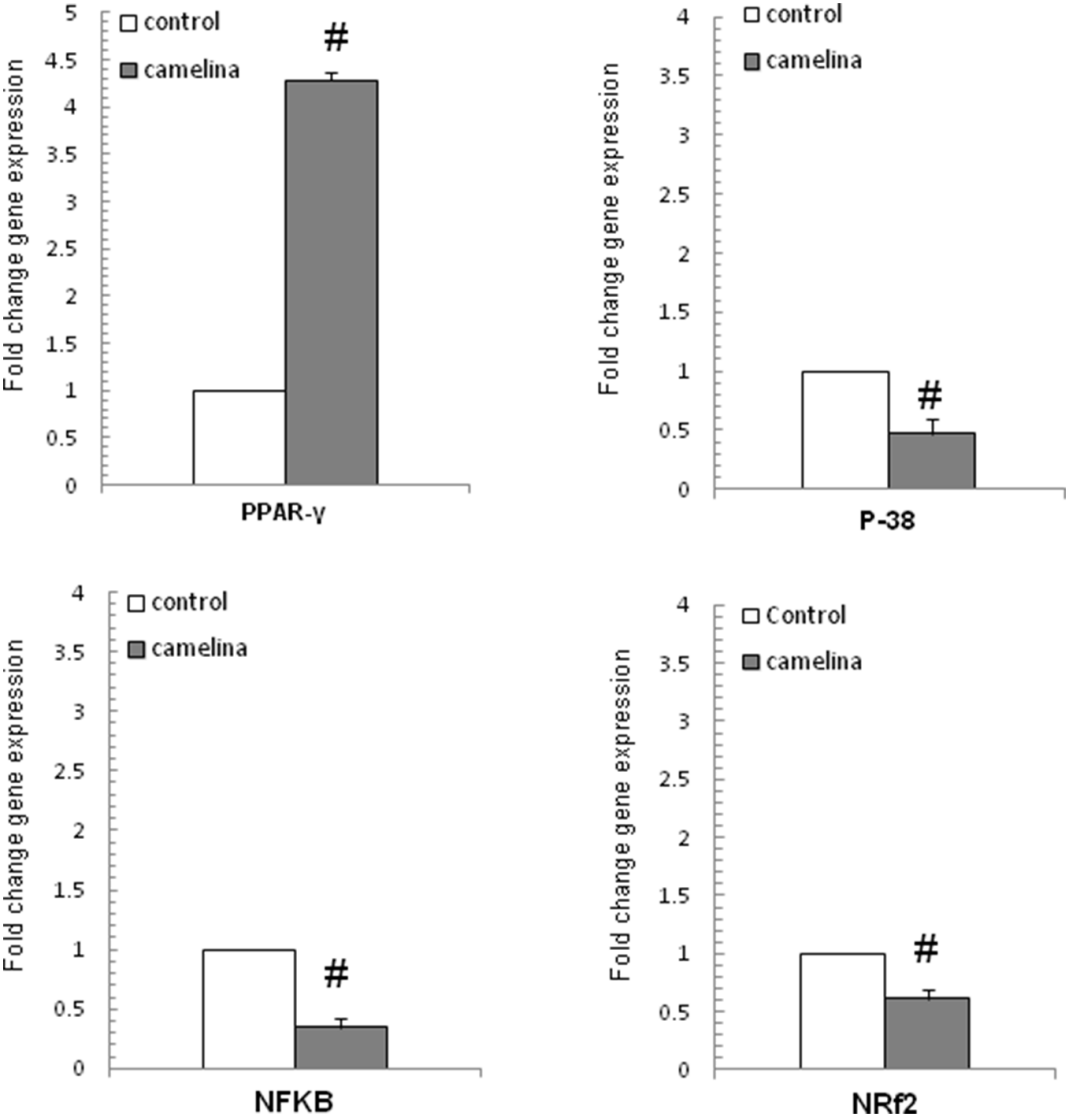
Effect of camelina oil-cakes on signaling molecules expression in spleen. Spleen samples were taken at the end of the trial on day 33 and were analyzed for PPAR-γ, NF-κB, MAPK-p-38α and Nrf2 mRNA expression by quantitative RT-PCR. Results are expressed as change after normalization of the expression of target gene to the mean of 2 internally reference genes expression. Values are the means ± SEM, from two replicates. Statistical analysis was performed using one-way ANOVA followed by Fisher test (*P<0.05, T1 diet-control group (white column) versus T2 diet -Camelina group (grey column).

Gene expression of nuclear factors PPARγ and NF-κB, MAPK-p38α and signaling molecules associated with cytokines synthesis and inflammation are presented in Figure 5. Our results showed a significant, 3.53 times increase of PPARγ in the spleen of pigs on the camelina oil-cakes diet (P<0.0001). Meanwhile, the expression of NF-κB and MAPK-p38α decreased by 1.41 and 3.83 (P<0.02) times, respectively.

**Figure 6:**
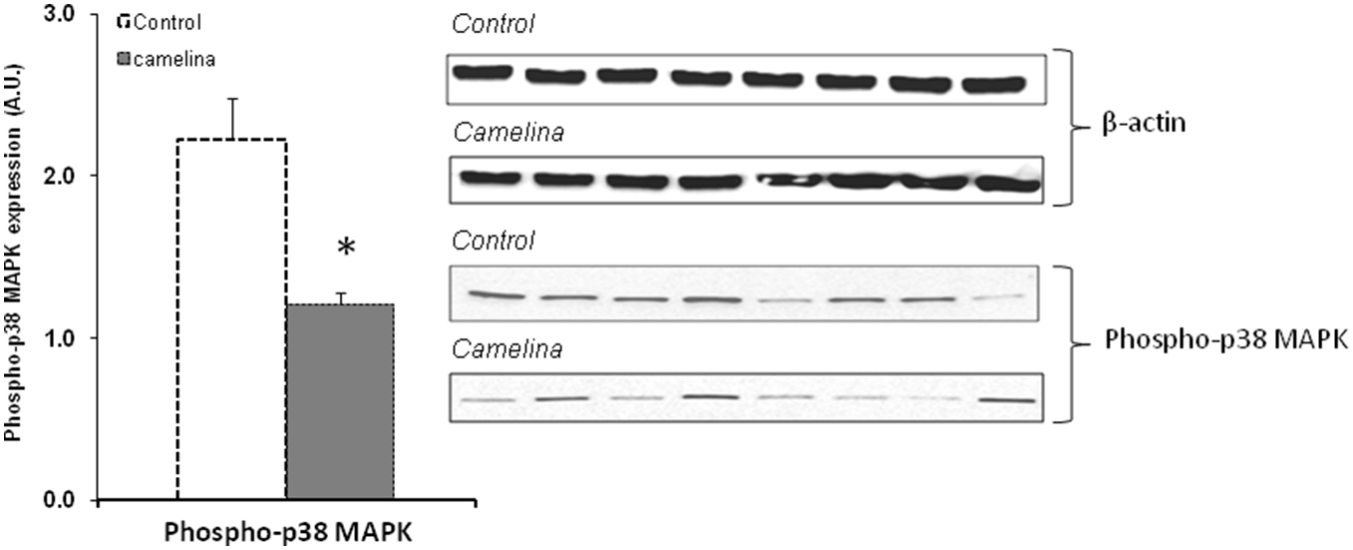
Phospho-MAPK-p38α expression in protein spleen lysate. The level of MAP-kinase p38α phosphorylation in spleen of pigs fed or not with camelina oil-cakes was determined by western blot and expressed as the ratio between phospho- MAPK-p38α and β-actin band intensities respectively. For each group of animals the mean values ± SEM were calculated and presented as histogram. Statistical analysis was performed using one-way ANOVA followed by Fisher test (*P<0.05, T1 diet-control group (white column) versus T2 diet -Camelina group (grey column).

**Figure 7:**
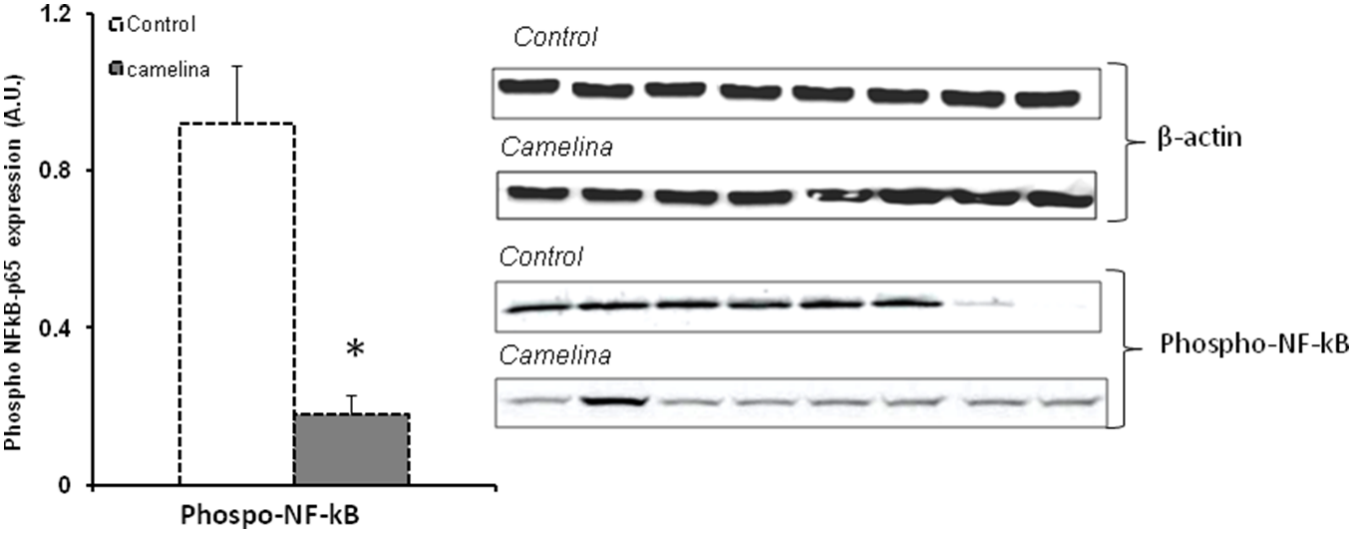
Phospho-p65 NF-κB expression in protein spleen lysate. The level of p65-NF-kB phosphorylation in spleen of pigs fed or not with camelina oil-cakes was determined by western blot and expressed as the ratio between phospho-p65 NF-κB and β-actin band intensities respectively. For each group of animals the mean values ± SEM were calculated and presented as histogram. Statistical analysis was performed using one-way ANOVA followed by Fisher test (*P<0.05, T1 diet-control group (white column) versus T2 diet -Camelina group (grey column).

The immunoblot analysis showed that the phosphorylated active forms of MAPK-p38α and NF-κB were significantly reduced (45.53% and 80.43% respectively, P<0.001) in the spleen samples collected from animals receiving the camelina oil-cakes diet (Figures 6 and 7).

### 7. Effect of the camelina oil-cakes on the antioxidant enzymes gene expression in spleen

**Figure 8:**
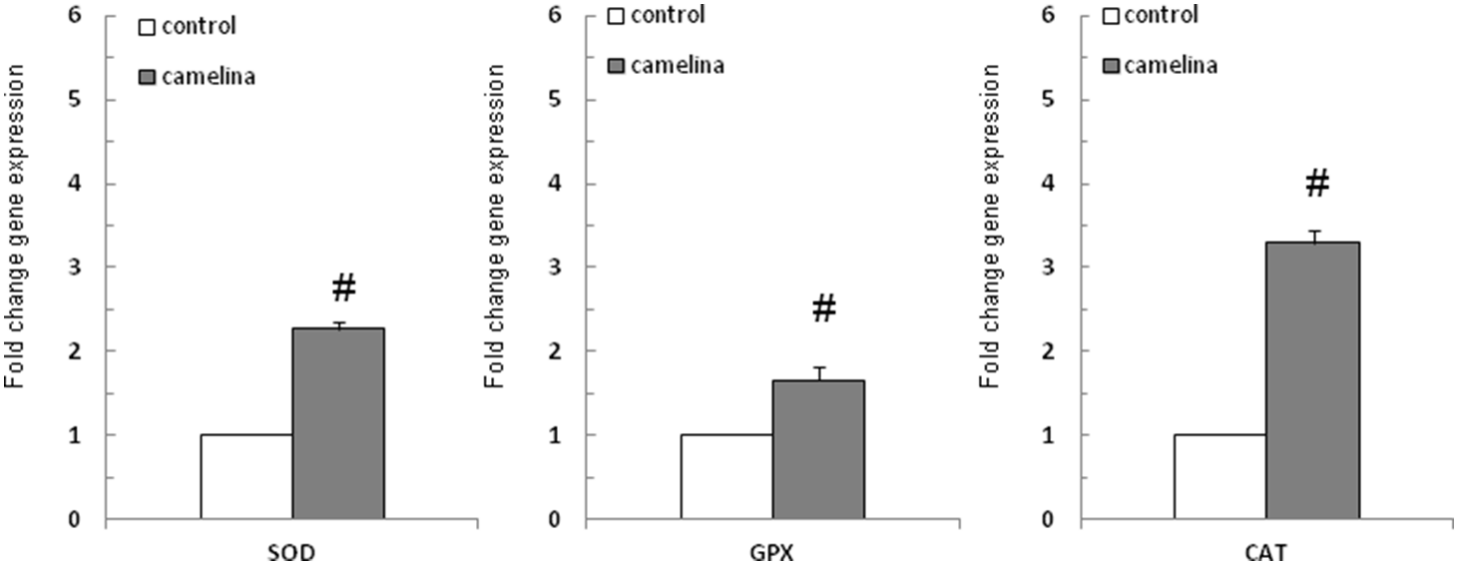
Effect of camelina oil-cakes on antioxidant enzymes expression. Spleen samples were taken at the end of the trial on day 33 and were analyzed for SOD, CAT and GPx mRNA expression by quantitative RT-PCR. Results are expressed as fold change after normalization of the expression of target gene to the mean of 2 internally reference genes expression. Values are the means ± SEM, from two replicates. Statistical analysis was performed using one-way ANOVA followed by Fisher test (*P<0.05, T1 diet-control group (white column) versus T2 diet -Camelina group (grey column).

The effect of the T2 diet on gene expression of antioxidant defense system components, such as CAT, SOD and GPx1, was assessed in the spleen. Results showed that mRNA expression of these enzymes increased significantly in the spleens of pigs fed camelina oil-cakes: 2.27 (SOD), 3.29 (CAT) and 1.66 (GPx1) times, respectively (Figure 8).

### 8. Correlations between gene expressions of nuclear receptors, signaling molecules, inflammatory related markers and antioxidant defense enzymes in the spleens of pigs fed on camelina oil-cakes

**Figure 9:**
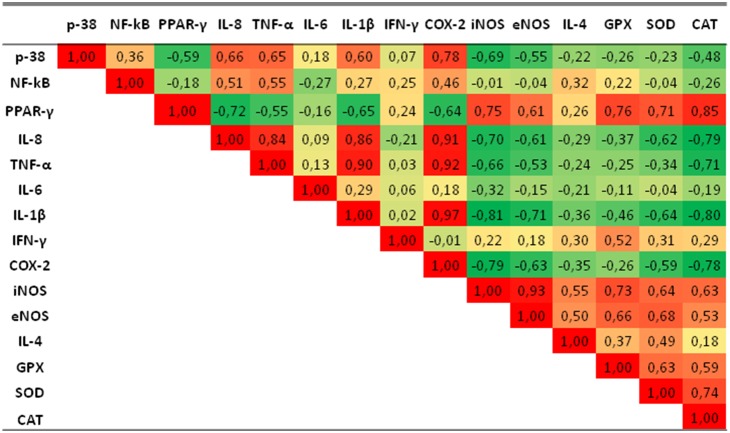
Correlations between gene expressions in spleen. Pearson correlation coefficient procedure was used to establish relationships between gene expression of nuclear receptors PPARγ, NF-κB and signalling p38-MAPK, inflammation-related molecules and antioxidant defense enzymes in spleen of pigs received dietary camelina cakes. The red and green colour gradient from dark to light shows the degree of positive or negative correlations respectively in spleen of pigs treated or not with camelina diet.

In order to better understand the mechanism of PUFA action, mathematical correlations were established between the expressions of nuclear receptors, signaling molecules, inflammatory related markers and antioxidant defense enzymes in spleen samples derived from pigs fed with T1 or camelina T2 diets. Highly significant negative correlations were obtained between PPAR-γ gene expression and the pro-inflammatory markers (IL-8: R^2^ = −0.72, TNF-α: R^2^ = −0.55 and IL-1β: R^2^ = −0.65) and also between PPAR-γ and the gene coding for COX-2 (R^2^ = −0.64). PPAR-γ was highly positively correlated with the expression of antioxidant enzymes (GPx: R2 = 076, SOD: R2 = 0.71 and CAT: R2 = 0.85). As expected, NF-κB and MAPK-p38α were negatively correlated with PPAR-γ and positively correlated with pro-inflammatory marker expression (TNF-α: R^2^ = 0.55; IL-8: R^2^ = 0.51). Also, negative correlations were found between the antioxidant enzymes and the pro-inflammatory markers (Figure 9).

## Discussion

Feeding a camelina oil-cakes diet to fattening pigs during the finishing period (33d) had no influence on feed intake, average weight gain, or feed efficiency. These results agree with other reports on camelina [17], [26]–[28], or other PUFA sources in monogastric animals or ruminants [9], [29]. However, similar to other PUFA studies, we found a significant decrease (P<0.05) of glucose concentration in the plasma of pigs fed on the camelina oil-cakes diet. Polyunsaturated fatty acids, especially ω-3 PUFA, are natural regulators of glucose uptake *in vivo,* being potential ligands and regulators for the PPAR-γ transcriptional factor, a member of the nuclear hormone receptors superfamily [30]. PPAR-γ in turn regulates gene expression and metabolic processes such as glycolysis, lipid biosynthesis, fatty acid elongation, desaturation, and oxidation [31]. It was shown that activators of PPAR-γ (i.e. thiazolidinediones) are largely used in the treatment of type 2 diabetes [32]. In this study, the T2 diet significantly increased mRNA expression of PPAR-γ and decreased glucose concentration in the blood plasma (84.24 *vs* 68.68 mg/dL, P = 0.018). Currently, PUFAs are used in the treatment of diabetes due to their potential to lower glucose levels in the blood, to promote a better glucose tolerance and to reduce the hazards associated with inflammation [30], [33]–[35]. However, Burri et al., (2011) affirmed that the effect on glucose level depends on the esterification form of ω-3 PUFAs, especially eicosapentaenoic acid (EPA) and docosahexaenoic (DHA) to either phospholipids or triglycerides, which could exert a different response [31]. For example glucose level was decreased by krill oil (ω-3 PUFAs esterified into phospholipids fraction) in mice and increased by canola oil in rat or fish oil in human [31], [36]–[38]. On the other hand no influence on plasma circulating glucose in pigs fed diets rich in ω-3 fatty acids was also observed [7], [39], [40]. It was suggested that the variable effect of fish oil on glycaemic control may be caused by variation in insulin sensitivity between subjects [41]. However, more studies are needed to elucidate the glucose lowering effect of ω-3 PUFAs in serum profiles. PUFAs are also involved in the lipid metabolism, acting differentially in their regulation (up or down) in accordance with the source of the PUFA [31]. Different effects of PUFAs at the metabolic level were also noticed for lipids [31], [42], [43]. In the present study, a trend towards a decrease in plasma cholesterol concentration during 33 days was identified in pigs fed with camelina oil-cakes.

Consumption of PUFAs is associated with modulatory effects on the expression and secretion of important markers of inflammation such as cytokines and chemokines in humans and pigs. For example, the intake of diets enriched with fish and flaxseed oils diminished pro-inflammatory cytokine (IL-1 and IL-6, and TNF-α) and adhesion molecule expression [44], [45] in humans, while studies carried out on mice revealed either a stimulatory or an inhibitory effect of ω-3 fatty acids on the pro-inflammatory cytokines [4]. Zhan and colleagues showed that a diet enriched with 10% linseed was able to linearly decrease (during the feeding period) the gene expression of these cytokines in muscle, spleen and adipose tissue in finishing pigs under normal physiological conditions [10]. In this study, feeding the 12% camelina oil-cakes (wild flaxseed) diet produced significantly less IL-1β, TNF-α, IL-6 and IL-8 at both the mRNA and protein level in the spleen, also under normal physiological conditions. Our results indicate that camelina oil-cakes by their active compounds, ω-3 fatty acids and other antioxidants (tocopherol, etc.) could modulate the shift between Th1/Th2 cytokine balance. It is a suppressor of Th1-type cytokines by decreasing the pro-inflammatory gene expression and an inducer of the Th2-type cytokines by increasing the IL-4 gene expression. The induction or suppression of one type or another of these cytokines might be exploited in different nutritional treatment strategies with important immunological consequences.

Based on literature data there are different mechanisms by which unsaturated fatty acids can suppress pro-inflammatory cytokine synthesis. ω-3 PUFA could exert their effects on these immune mediators by acting directly on the intracellular signalling pathways, resulting in activation or inactivation of several nuclear transcriptional factors (PPAR-γ, NF-κB) involved in the regulation of the immune response, particularly in inflammation [10], [46], [47]. In our study, a significant increase of the expression of PPAR-γ gene was observed in the spleen of pigs fed camelina oil-cakes. A stimulatory effect of a flaxseed diet on PPAR-γ gene was also found by Zhan and colleagues in finishing pigs [10]. These authors reported negative correlations between the expression of PPAR-γ and the expression of pro-inflammatory cytokines in muscle and spleen and suggested that dietary ω-3 PUFA from flaxseed might inhibit pro-inflammatory mediators by activating PPAR-γ. PPAR-γ is a member of the nuclear hormone receptors superfamily which regulates immune response by repressing NF-κB signalling and inflammatory cytokine production [7]. In our study, significant negative linear correlations were found between the expression of PPAR-γ and the expression of inflammatory cytokines. A decrease in the expression of NF-κB and MAPK-p38α was also observed in the spleen of pigs fed PUFAs from camelina oil-cakes. Recent studies [8] indicate that NF-κB inhibition by PUFAs could also be mediated by the activation of G-protein coupled receptor 120 (GPR 120) which functions as a PUFA receptor. The stimulation of GPR120 inhibits TAK-1 (transforming growth factor-β-activated kinase-1), an upstream activator of MAPK pro-inflammatory signalling pathways (JNKs and MAPK-p38α), and of NF-κB, thereby repressing tissue inflammation. Furthermore, some ω-3 fatty acids could influence the activity of nuclear receptors by affecting their phosphorylation state [9], [49], [50]. Our immunoblot analysis showed that the phosphorylation level of MAPK-p38α and NF-κB was significantly reduced (54.47%, *P*<0.06, and 19.57% respectively) in spleen samples collected from animals receiving dietary camelina oil-cakes (Figure 6 and Figure 7) compared to the control.

The above mentioned authors suggested that ω-3 PUFA may have an anti-inflammatory effect through the activation of the vague nerve which leads to the inhibition of NF-κB and activation of STAT3 molecule. Phosphorylation of STAT3 increases the expression of SOCS3 (suppressor of cytokine signalling 3) which in turn inhibits cytokine synthesis [48]. These mechanisms mainly occur in macrophages, which are abundant in the spleen. However, more studies are needed to elucidate these mechanisms.

PUFAs affect the expression and activation of many other inflammatory mediators [51]. As expected, qPCR data identified a significant decrease of COX2 gene expression under the action of the camelina diet and, interestingly, a significant increase in iNOS and eNOS mRNA. Most studies with unsaturated fatty acids have indicated a decrease in iNOS expression and NADPH activity [52], [53], but this specific regulatory effect is not shared by all fatty acids. Some of them and other nutritional phytochemicals are COX2 inhibitors and iNOS activators [54]. For example, the arachidonic (ω-6) and eicosapentaenoic (ω-3) fatty acids, Echinaceea extract and several phenols induced an increased iNOS gene expression [55], [51], [56]. It was suggested that the underlying mechanism is independent of COX and NF-κB pathway and cytokines (IL-1, TNF-α and IFN-γ), being mediated instead by protein kinase C and tyrosine kinase [55]. By contrast, the regulation of iNOS transcription is mediated by increased NO through negative feedback, which inhibits NF-κB binding to DNA [51]. An increased spleen synthesis of NO was also produced by camelina oil-cakes diet in our study.

The effects of PUFA on oxidative stress have not been extensively studied [39]. According to some researchers, diets rich in fish oil could cause oxidative damage in humans and animals due to a high level of unsaturation in the PUFAs molecular structure, while others demonstrated that dietary fish oil has antioxidant effects [33]. For example, plasma antioxidant capacity and the activity of the major antioxidant enzymes SOD, GPx1, CAT, were increased by dietary ω-3 PUFA (especially EPA and DHA) in rats [57], [58], [59]. These results concur with those observed in the present study, in which we found a significant increase in mRNA expression of SOD, CAT, GPx1 and in total plasma antioxidant capacity. Investigating the capacity of a rich ω-6/ω-3 PUFA fermented wheat powder (Lisosan G) to modulate antioxidant and detoxifying enzymes, La Marca and colleagues [60] found an increase in gene expression and activity of several antioxidant/detoxifying enzymes, and identified the underlying molecular mechanism that determined the antioxidant properties of the unsaturated fatty acids related to the activation of Nrf2 and to the inhibition of NF-κB [60]. Meadus and colleagues [19] suggested that the increase of phase 2 xenobiotic detoxifying enzymes CyPb1, Aldh2, TST and GstM1 gene expression in pig liver was induced by camelina meal through its glucosinolates, glucocamelina (methyl-sulfinyldecyl isothiocyanates) which is able to induce Nrf2 activation [19]. Nrf2 is a transcriptional factor playing an essential role in the induction of antioxidant enzymes that confer protection against oxidative stress [61], [62]. Many examples showed that Nrf2 and NF-κB pathways interfere in controlling the transcription or function of downstream target proteins. Cross talk between different members of these two protein families range from direct effects on transcription factors to protein-protein interactions [63], [60]. It has been shown in rats that a diet supplemented with multiple antioxidants reduced the increase in oxidative stress with concomitant inhibition of NF-κB [64]. On the other hand, it was shown that the overexpression of antioxidant enzymes, other antioxidants or pharmacological inhibitors of NF-κB and MAPK-p38α inhibits the expression of Nrf2 [65]. In our study, the gene expression of Nrf2 was significantly decreased by the camelina diet in spleen.

It was proved that the humoral immune response is modulated by ω-3 PUFA. For example, dietary supplementation with fish oil decreased antibody production in mice [66] and humans [67]. However, Chang and colleagues [68] reported that ω-3 PUFA oils increased nonspecific IgE level in BALB/c mice serum, and lowered nonspecific IgA and OVA-specific IgG1 level [64]. Anti-OVA antibody concentration was higher in sows and their piglets fed dietary flaxseed and flaxseed meal than in the animals fed a diet supplemented with flaxseed oil [7], suggesting that the effect can differ according to the dietary form: seed, cakes, meal or oil. In the present study no effect on IgA, IgM and IgG was produced by the camelina diet.

### Conclusion

Our results taken together indicate that the diet including camelina oil-cakes did not influence pig performance, but modulated several mediators of the cellular immune response (decrease of pro-inflammatory cytokines and COX2), antioxidant defense system (increase in antioxidant enzymes expression, SOD, CAT, GPx, and NO production) in the spleen. Also, the camelina oil-cakes diet improved the blood biochemistry profile: decrease in plasma glucose and increase of plasma antioxidant capacity. All these results indicate that camelina oil by-products rich in ω-3 PUFA have the capacity to modulate systemic metabolism and to influence spleen cell function. Thus, *Camelina* could be an alternative to other oilseeds as a source of ω-3 PUFA and other antioxidants, which can be further modulated by specific dietary strategies. At this level of inclusion (12%) camelina oil-cakes appear to be a potentially alternative source of fat for pigs, preserving a high content of ω-3 PUFA. Its antioxidant potential is shown by the stimulation of detoxifying enzyme gene expression and by the suppression of pro-inflammatory markers in spleen. Moreover, the recent research evidences in pigs, demonstrated that some of camelina constituents (erucic acid and glucosinolates) considered as anti-nutritional factors which limit its utilisation might have anti-carcinogenic benefits, by stimulating the hepatic expression of phase 1 and 2 xenobiotic detoxifying enzymes.
